# Evolution of group I introns in Porifera: new evidence for intron mobility and implications for DNA barcoding

**DOI:** 10.1186/s12862-017-0928-9

**Published:** 2017-03-20

**Authors:** Astrid Schuster, Jose V. Lopez, Leontine E. Becking, Michelle Kelly, Shirley A. Pomponi, Gert Wörheide, Dirk Erpenbeck, Paco Cárdenas

**Affiliations:** 1 Department of Earth- & Environmental Sciences, Palaeontology and Geobiology, Ludwig-Maximilians-Universität München, Richard-Wagner-Str. 10, 80333 Munich, Germany; 2 Halmos College of Natural Sciences and Oceanography, Nova Southeastern University, Dania Beach, FL 33004 USA; 3 Marine Animal Ecology, Wageningen University & Research Centre, P.O. Box 3700, AH, Wageningen, The Netherlands; 4 Naturalis Biodiversity Center, Marine Zoology Department, PO Box 9517, 2300 RA, Leiden, The Netherlands; 5 National Centre for Aquatic Biodiversity and Biosecurity, National Institute of Water and Atmospheric Research, P.O. Box 109–695, Newmarket, Auckland, New Zealand; 6 Harbor Branch Oceanographic Institute-Florida Atlantic University, 5600 U.S. 1 North, Ft Pierce, FL 34946 USA; 7 SNSB - Bavarian State Collections of Palaeontology and Geology, Richard-Wagner Str. 10, 80333 Munich, Germany; 8 GeoBio-CenterLMU, Ludwig-Maximilians-Universität München, Richard-Wagner Str. 10, 80333 Munich, Germany; 9 Department of Medicinal Chemistry, Division of Pharmacognosy, BioMedical Center, Uppsala University, Husargatan 3, 75123 Uppsala, Sweden

**Keywords:** Porifera, Tetractinellida, *cox1*, HGT, VGT, homing endonuclease gene (HEG), LAGLIDADG, group I intron, DNA barcoding

## Abstract

**Background:**

Mitochondrial introns intermit coding regions of genes and feature characteristic secondary structures and splicing mechanisms. In metazoans, mitochondrial introns have only been detected in sponges, cnidarians, placozoans and one annelid species. Within demosponges, group I and group II introns are present in six families. Based on different insertion sites within the *cox1* gene and secondary structures, four types of group I and two types of group II introns are known, which can harbor up to three encoding homing endonuclease genes (HEG) of the LAGLIDADG family (group I) and/or reverse transcriptase (group II). However, only little is known about sponge intron mobility, transmission, and origin due to the lack of a comprehensive dataset. We analyzed the largest dataset on sponge mitochondrial group I introns to date: 95 specimens, from 11 different sponge genera which provided novel insights into the evolution of group I introns.

**Results:**

For the first time group I introns were detected in four genera of the sponge family Scleritodermidae (*Scleritoderma, Microscleroderma, Aciculites, Setidium*). We demonstrated that group I introns in sponges aggregate in the most conserved regions of *cox1*. We showed that co-occurrence of two introns in *cox1* is unique among metazoans, but not uncommon in sponges. However, this combination always associates an active intron with a degenerating one. Earlier hypotheses of HGT were confirmed and for the first time VGT and secondary losses of introns conclusively demonstrated.

**Conclusion:**

This study validates the subclass Spirophorina (Tetractinellida) as an *intron hotspot* in sponges. Our analyses confirm that most sponge group I introns probably originated from fungi. DNA barcoding is discussed and the application of alternative primers suggested.

**Electronic supplementary material:**

The online version of this article (doi:10.1186/s12862-017-0928-9) contains supplementary material, which is available to authorized users.

## Background

Mobile introns are self-splicing DNA sequences that play a major role in genome evolution. Group I and group II introns are distinguished based on their splicing mechanisms and secondary structures. Apart from unique splicing mechanisms, differences between group I and group II introns were observed within the core regions of their secondary structures. Depending on these structural characteristics, group I introns have been further categorized into IA-IE classes. Group II introns constitute up to six stem-loop domains and are classified I-VI respectively (e.g., [35]). Group I and group II introns often contain open reading frames (ORFs) in their loop regions [70], which can encode for different site-specific homing endonuclease genes (HEGs). The majority of group I introns include HEGs, which have a conservative single or a double motif of the amino-acid sequence LAGLIDADG. In contrast group II introns encode in most cases a reverse transcriptase-like (RT) ORF (e.g., [36]). Group I and group II introns are found in all domains of life: group I introns are present in bacterial, organellar, bacteriophage and viral genomes as well as in the nuclear rDNA of eukaryotes. Group II introns have a similar distribution, but are not known from the nuclear rDNA (e.g., [33]). More specifically, group I and/or group II introns are found, e.g., in eukaryotic viruses [92], slime molds [45], choanoflagellates [7], the annelid *Nephtys* sp. [84], red algae [8], brown algae [25] and plants: green algae [85], liverworts [58, 66, 67] and different angiosperms [58, 66, 67]. Group II introns seem to thrive especially in plants [60], whereas the largest abundance of group I introns currently occurs within fungi [23, 54, 68]. As an example, the mitochondrial (mt) genome of the fungus *Ophioscordyceps sinensis* harbors 44 group I introns and six group II introns, accounting for 68.5% of its mt genome nucleotides. Here, 12 out of 44 group I introns and only one out of six group II introns are located in the cytochrome c oxidase subunit 1 (*cox1*) gene [54], an acknowledged insertion hotspot for mt group I introns [23].

**Fig. 1:**
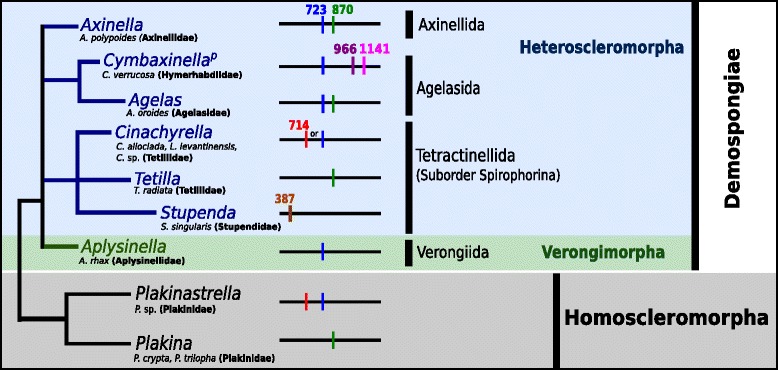
Simplified sponge phylogeny highlighting currently known group I and II intron insertion sites. Horizontal *black* lines with colored vertical bars and numbers (*blue*: intron 723, *green*: intron 870, *red*: intron 714, *brown*: intron 387, *purple*: intron 966, *pink*: intron 1141) behind the taxa names represent different intron locations within *cox1*

More recently, group I introns have been discovered in the *cox1* of early branching metazoan phyla: Placozoa [9, 17, 76], Cnidaria [27, 31] as well as Porifera [21, 29, 65, 82, 90]. Group II introns are rarer, and found in the *cox1* of Placozoa [17, 76], and in one demosponge species of the order Axinellida (referred to as *Cymbaxinella verrucosa*) [43]. In Porifera, group I introns have only been recorded from Demospongiae and Homoscleromorpha and, like in group II introns, always in the *cox1* gene, with only occasional double insertions (Fig. 1). The current nomenclature of sponge group I and group II introns is based on the intron insertion site positions in reference to the *Amphimedon queenslandica cox1* gene (DQ915601) [82]. In Homoscleromorpha, three different intron positions (714, 723 and 870; Fig. 1) are known for three species of the family Plakinidae [29]. Within the demosponge subclass Verongimorpha intron 723 is detected in one species (*Aplysinella rhax*) of the order Verongiida [21]. Most intron insertions have been found within the demosponge subclass Heteroscleromorpha, in the orders Agelasida, Axinellida [43] and especially Tetractinellida [82]. In the Tetractinellida, group I introns are currently known in five sponge species belonging to three genera (*Cinachyrella*, *Tetilla* and *Stupenda*) and inserted at four mtDNA intron positions: 387, 714, 723 and 870 [47, 65, 82] (Fig. 1). To this date, all sponge group I introns encode a HEG with two LAGLIDADG motifs [21, 29, 47, 82] with the exception of intron 714 in *Plakinastrella* sp. and intron 870 in *Agelas oroides* and *Axinella polypoides,* in which no ORF was detected [29, 43, 52, 90]. Intriguingly, Tetractinellida introns are currently only detected in the families Tetillidae [82] and Stupendidae [47].

Fungi and Placozoa have been proposed as possible donors for group I introns among sponges [43, 47, 65]. However, these findings await corroboration with a broader and more comprehensive taxon set. Intron/HEG phylogenetic analyses and group I/II intron secondary structures are the basis for different scenarios on the origin of introns within sponges [43, 47, 82]. The presence of independent horizontal gene transfers (HGT) for introns is supported by their haphazard distribution over phylogenetically distant sponge groups [21, 43, 82]. Vertical gene transfer (VGT) of introns is assumed among closely related taxa, but never confirmed due to the lack of comprehensive taxon sampling [82].

To gain new insights into the evolution of sponge introns we required an intron-rich taxonomic group. Based on earlier studies on sponge introns, the order Tetractinellida represents an obvious target. Other lines of evidence support this choice, such as unsuccessful attempts to amplify *cox1* in this group with standard protocols [10, 72, 81], potentially due to introns in the relevant primer regions [82]. Consequently, this study focuses on tetractinellid *cox1* mitochondrial data to broaden our knowledge on mt intron evolution in this early-branching metazoan phylum.

The data from this “intron-hotspot taxon” presented here constitutes the most representative dataset to target specific questions pivotal to understand intron structure and distribution including activity and mobility. Importance of HGT or VGT or a combination of both will be addressed. Additionally, current hypotheses on the origin of sponge mitochondrial introns will be discussed by comparing intron data across other phyla.

## Results

### Mitochondrial intron diversity and characteristics in tetractinellid sponges

**Fig. 2:**
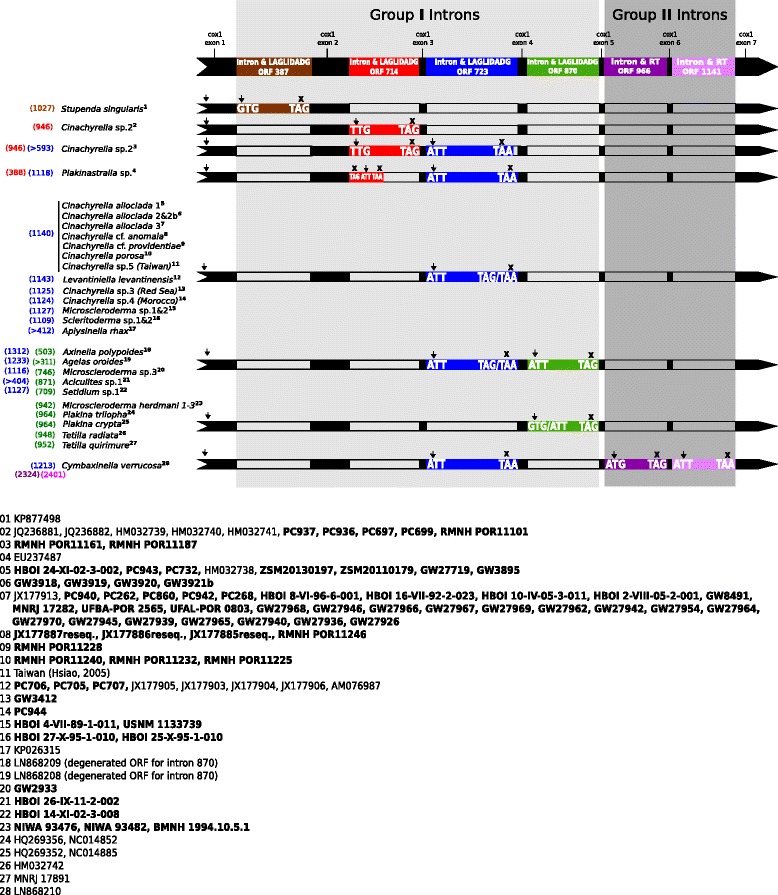
Overview of different *cox1* intron positions in sponges. Four group I introns (387, 714, 723, 870) and two group II introns (966 + 1141) are distinguished and labeled by colors according to their different insertion sites. Arrows and Xs above each intron insertion indicate start and stop codons respectively. As not all taxa have the same start codon, both possible start codons are given. Numbers to the left of species names refer to sequence lengths of each intron (in bp), they match the intron color. Numbers to the right of species names (not the superscript) refer to unresolved species complexes

The current study comprises the largest dataset of sponge mitochondrial introns to date (95 sequences of which 72 are new), encompassing 13 different sponge genera. All 72 newly sequenced introns were group I introns of the class IB, and all encoded a HEG of the LAGLIDADG family, except for intron 723 of *Aciculites* sp.1, where no HEG was observed. A double motif of the LAGLIDADG domain was located in all introns, if the sequence was not degenerated or without a HEG. Different intron lengths were observed for different species, and an overview of the different initiation and stop codons of all HE ORFs is given in Fig. 2. All introns possessed start and stop codons in the same frame as the 5′ exon, except intron 714 in *Plakinastrella* sp. Additionally, uninterrupted ORFs in the same 5′ exonic reading frame were observed for all sequences unless introns were degenerated or without HEG. Initiation and stop codons varied among the intron HEGs. For example the HEG of intron 387 potentially starts with a GTG initiation codon at position 19 of the intron, and not TTA (position 16) as suggested previously [47]. The HEG of intron 714 potentially starts with TTG as its initial codon (position 27). Instead of TTA (position 1) as suggested by Rot et al. [65], all intron 723 HEGs potentially start with ATT (position 10). In the intron HEG 870 we have ATT (position 24 for all) and GTG (position 9 for *Plakina* and 21 for *Tetilla*) as initiation codons. The stop codon for most HE ORFs was TAG or TAA except for *Cinachyrella* sp. 2 and *Setidium* sp.1 (intron 723), where truncated HEGs were found.

We discovered more introns in the Spirophorina at positions 714, 723 and 870 (Fig. 2); no intron at position 387 was found. As an example, intron 714 sequences were generated for five more *Cinachyrella* sp. 2 taxa; four from the Indian Ocean (Kenya, Myanmar) and one from the southwest-Pacific (Indonesia). *Cinachyrella* species are previously known to have only one intron insertion at a time (either 714 or 723). However, our study reveals that both introns 714 and 723 can occur together in *cox1*, e.g., in *Cinachyrella* sp. 2 from marine lakes (RMNH POR11161) and mangroves (RMNH POR11187). Intron 723 was sequenced from 11 different *Cinachyrella* species, and it is particularly present in the *Cinachyrella alloclada* complex. In total this study contains 42 sequences of *Cinachyrella alloclada* (intron 723) from the western Atlantic, the Caribbean Sea and the Gulf of Mexico. We added six additional intron 723 sequences including *C*. cf. *anomala*, *C*. cf. *providentiae*, *C. porosa* (all Indonesia), *C*. sp. 3 (Red Sea), *C*. sp. 4 (Morocco) and *C.* sp. 5 (Taiwan). The resulting *Cinachyrella* dataset covers subtropical-tropical areas from 1 to 90 m depth.

For the first time we discovered intron 723 in the Scleritodermidae (*Microscleroderma, Aciculites, Setidium* and *Scleritoderma*). Intron 870 was found in *Tetilla quirimure* from Brazil and *Microscleroderma herdmani* from the Indian Ocean (Mauritius), and the Pacific (Philippines and Hawaii). Huchon et al. [43] located intron 723 in combination with intron 870 in two families (Axinellidae and Agelasidae), while our study reveals this combination in three scleritodermid genera (*Microscleroderma*, *Aciculites*, *Setidium*).

### Comparative intron and exon phylogenies of Tetractinellida

Phylogenetic reconstructions of the *cox1* exon and the intron revealed a patchy distribution of intron insertions among the Scleritodermidae and Tetillidae and different levels of congruence among intron and exon phylogenies.

#### Family Tetillidae

**Fig. 3:**
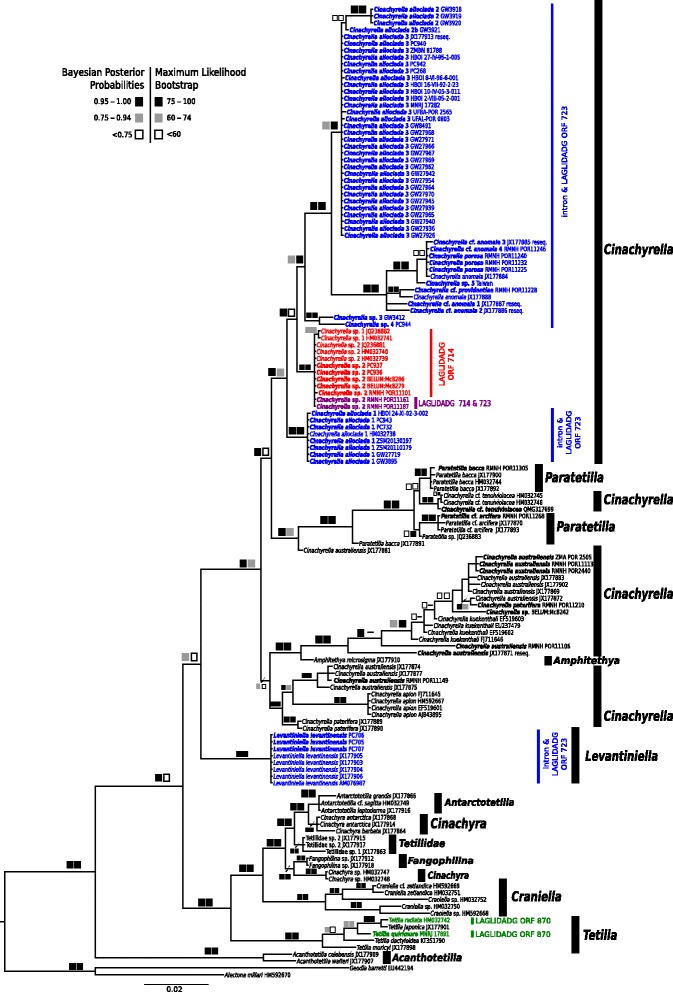
*Cox1* (introns excluded) bayesian inference (MrBayes, GTR + G + I model) phylogeny of the family Tetillidae. The maximum likelihood (RAxML) tree is congruent. Squares represent node supports. *Black* squares: PP = 0.95–1.00, BP = 75–100. *Dark gray* squares: PP = 0.75–0.94, BP = 60–74. *White* squares: PP < 0.75, BP < 60. Numbers behind each taxa are GenBank accession numbers or voucher numbers (for new sequences). Sequences generated in this study are in bold. Color code follows Fig. 2 and corresponds to the different intron insertion positions, except two *Cinachyrella* sp. 2 species, which are marked in *purple* as they possess two introns (714 + 723)

The relationships of major clades (Fig. 3) were in concordance with a previous study [81]. Unlike *Cinachyrella*, *Tetilla* appeared monophyletic in reconstructions with five species (*T. radiata*, *T. japonica*, *T. quirimure*, *T. dactyloidea* and *T. muricyi*). *Tetilla radiata* (intron 870) is sister to the intron-lacking *Tetilla japonica* (posterior probability [PP] = 1.00 / bootstrap support [BS] = 98). As shown by Szitenberg et al. [81], our analysis supported the early-branching position of the intron 723-bearing *Levantiniella levantinensis* with respect to the *Cinachyrella/Paratetilla/Amphitethya* clade. Intron 723-bearing Tetillidae did not form a monophyletic group due to the position of *L. levantinensis* and the presence of a highly supported (PP 1.00/BS 100) clade of intron 714 bearing species consisting of *C*. sp. 1 (intron 714 only) and *C*. sp. 2 (with intron 714 only or in combination with intron 723). There were no genetic differences in *cox1* between *Cinachyrella* sp. 2 bearing one (714) or two (714 *and* 723) introns.

#### Family Scleritodermidae

**Fig. 4:**
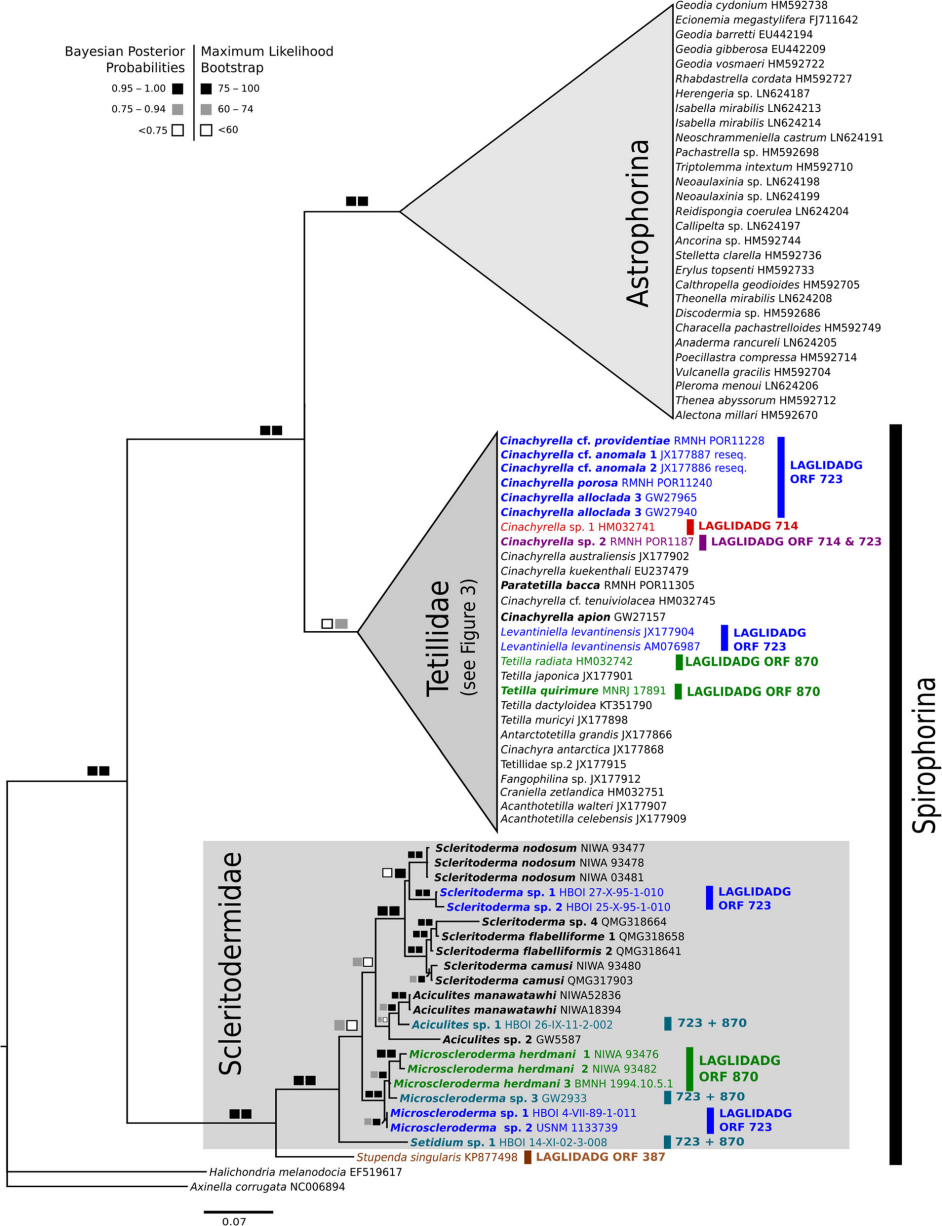
*Cox1* (introns excluded) bayesian inference (MrBayes, GTR + G + I model) phylogeny of the Tetractinellida with focus on the family Scleritodermidae. The maximum likelihood (RAxML) tree is congruent. Squares represent node supports. *Black* squares: PP = 0.95–1.00, BP = 75–100. *Dark gray* squares: PP = 0.75–0.94, BP = 60–74. *White* squares: PP < 0.75, BP < 60. Numbers after each taxa are GenBank accession numbers or voucher numbers (for new sequences). Sequences generated in this study are in bold (32 sequences). Color code follows Fig. 2, except for three Scleritodermidae taxa, which possess two intron insertions (723 + 870) and marked in *dark-cyan* respectively

The *cox1* exon phylogeny (Fig. 4) corroborated the sister group relationship Scleritodermidae/Stupdendidae, previously suggested with 18S rDNA data [47]. Results also supported the monophyly of Scleritodermidae and its genera *Microscleroderma, Aciculites* and *Scleritoderma* as previously suggested by 18S and 28S rDNA phylogenies [61, 72]. Species in these genera displayed different intron distributions (Fig. 4).

#### Intron + LAGLIDADG Phylogeny

**Fig. 5:**
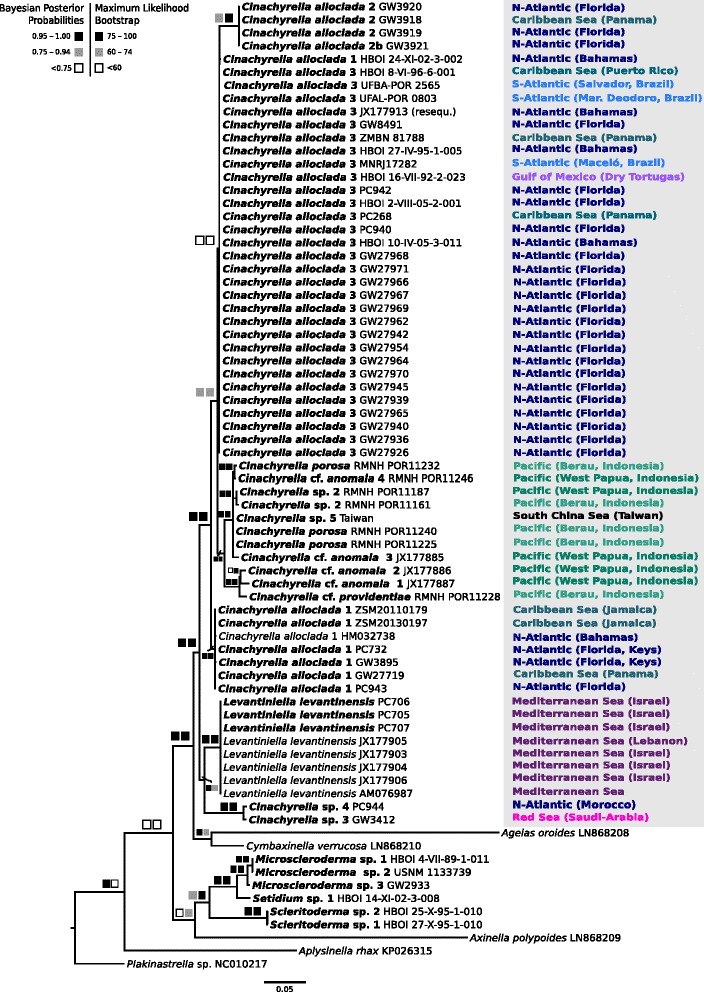
Intron 723 bayesian inference (MrBayes, GTR + G + I model) phylogeny in sponges. The maximum likelihood (RAxML) tree is congruent. Squares represent node supports. *Black* squares: PP = 0.95–1.00, BP = 75–100. *Dark gray* squares: PP = 0.75–0.94, BP = 60–74. *White* squares: PP < 0.75, BP < 60. Numbers after each taxa are GenBank accession numbers or voucher numbers (cf. Additional file 1). Sequences generated in this study are in bold (63 sequences). Sampling localities for the subtropical-tropical *Cinachyrella* taxa are given

The intron 723 phylogeny (intron + LAGLIDADG) (Fig. 5) broadly agreed with the corresponding exon phylogeny (Fig. 3), but also displayed several differences crucial for the understanding of sponge intron evolution. Notably, we recovered intron 723 of *Cinachyrella* sp. 2 in a different clade than for the *cox1* of all *Cinachyrella* sp. 2, whether they have intron 714 or both 714 + 723 (Fig. 3). The clade of introns 723 of *Cinachyrella* sp. 3 and sp. 4, from the Red Sea and Morocco respectively, were in a sister-group relationship with *L. levantinensis* whereas in the exon phylogeny these two species branched within the *Cinachyrella* clade (Fig. 3). These incongruences between the exon and the intron phylogenies were shown to be significant (*p* < 0.01, Shimodaira-Hasegawa (SH)-test). One single *C. alloclada* 1 sequence falls within the *C. alloclada* 3 clade. This position is regarded as artifactual due to an incomplete intron sequence as retrieved from degraded DNA.

Interestingly, *Agelas oroides* and *Cymbaxinella verrucosa* intron sequences grouped within Spirophorina in a highly supported sister group to *Cinachyrella* introns. The intron + LAGLIDADG phylogenies for 870 and 714 were congruent with the exon phylogeny for all supported clades (Additional file 1).

#### Secondary structure analyses of introns 723 and 870

**Fig. 6:**
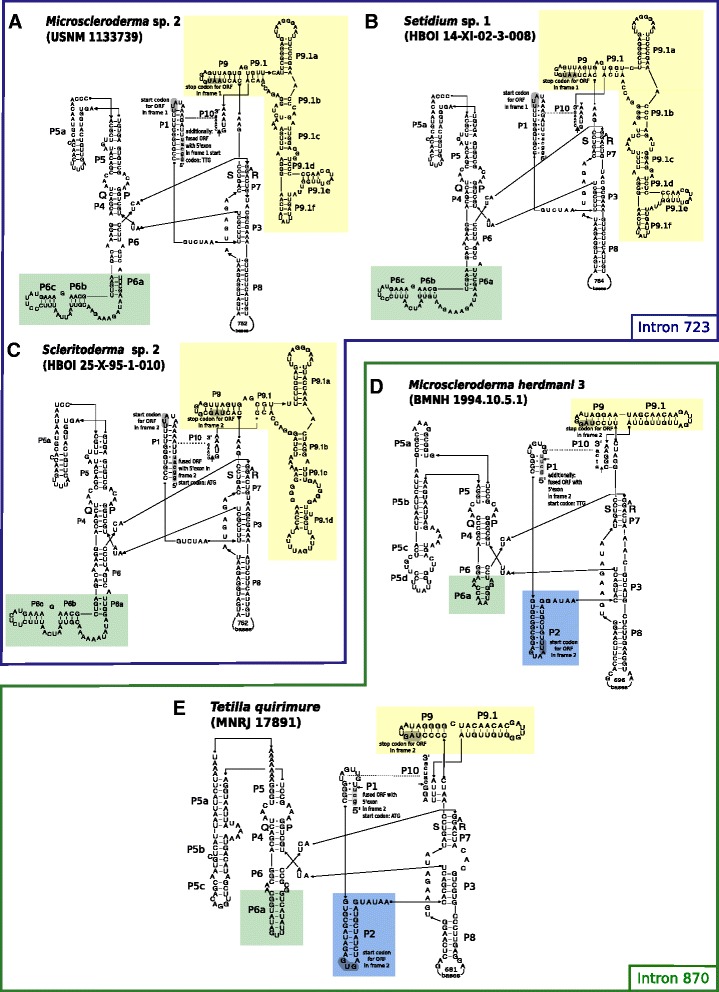
Predicted secondary structures of *Microscleroderma* sp. 2 (**a**), *Setidium* sp. 1 (**b**), *Scleritoderma* sp. 2 (**c**) (group I, IB, intron 723), *Microscleroderma herdmani* 3 (**d**) and *Tetilla quirimure* (**e**) (group I, IB, intron 870). Exon bases are in lower-case letters and intron bases in upper-case letters. Paired P1-P10 helices and their conserved sequences (P, Q, R, S) are labeled according to the standard group I intron scheme [88]. The HEGs are present in the loops of their respective P8 helix. For a better comparison the same color scheme as in Szitenberg et al. [82] was used to highlight differences in the P2, P6 and P9 regions. Potential start and stop codons are highlighted in *light gray*

The secondary structures of intron 723 and intron 870 presented the typical RNA fold of a group I intron structure [88], consisting of a P1-P2-P10 substrate domain, a P4-P5-P6 scaffold domain and a P3-P7-P8 catalytic domain (Fig. 6). The conserved regions Q, P, S, and R, building the core, were found in all of the structures.

Our current study expands our knowledge on *Cinachyrella* intron 723 [82] thanks to five additional structures predicted for *Cinachyrella porosa* (RMNH POR11225), *Cinachyrella* sp. 4 (PC944), *Cinachyrella alloclada* 2 (GW3920), *Cinachyrella* cf. *providentiae* (RMNH POR11228), and *Cinachyrella* cf. *anomala* (JX177887) (Additional file 2). The structures of introns 714 and 723 only have a single-stranded P2 region, whereas intron 870 has a double stranded P2 region (Fig. 6, [82]). The LAGLIDADG ORF is always located in the loop of the P8 helix (Fig. 6, Additional file 2).

Intron 723 structural differences between the species are in the P6 and P9 regions. In particular, *Cinachyrella* sp. 4 from Morocco has reduced helices P9.1c and P9.1d compared to all others. *Cinachyrella alloclada* 2 differs slightly in the P6, P6a, P6b and P6d regions to other *Cinachyrella* species. We generated for the first time secondary structures of scleritodermid intron 723 in *Microscleroderma* sp. 2 (USNM 1133739), *Setidium* sp. 1 (HBOI 14-XI-02-3-008) and *Scleritoderma* sp. 2 (HBOI 25-X-95-1-010) (Fig. 6). All three species show a high variability in loops and helices within the P9 region. Only a few differences were observed in the P6 region between the species. The main difference between *Cinachyrella* and Scleritodermidae intron 723 is the absence of the P6d region in the latter (Fig. 6a, b and c).

The secondary structure of intron 870 was reconstructed for *Tetilla quirimure* (MNRJ 17891) and *Microscleroderma herdmani* 3 (BMNH 1994.10.5.1) (Fig. 6d and e). Both taxa contain the known core helices and conserved structures of Q, P, S, and R. *Tetilla quirimure* intron structure is very similar regarding P6 and P9 regions to the one from *Tetilla radiata* [82]. The intron of *Microscleroderma herdmani*, in turn, has a reduced P6a helix and a different P5a region compared to that found in *Tetilla*.

#### The LAGLIDADG protein phylogeny

**Fig. 7:**
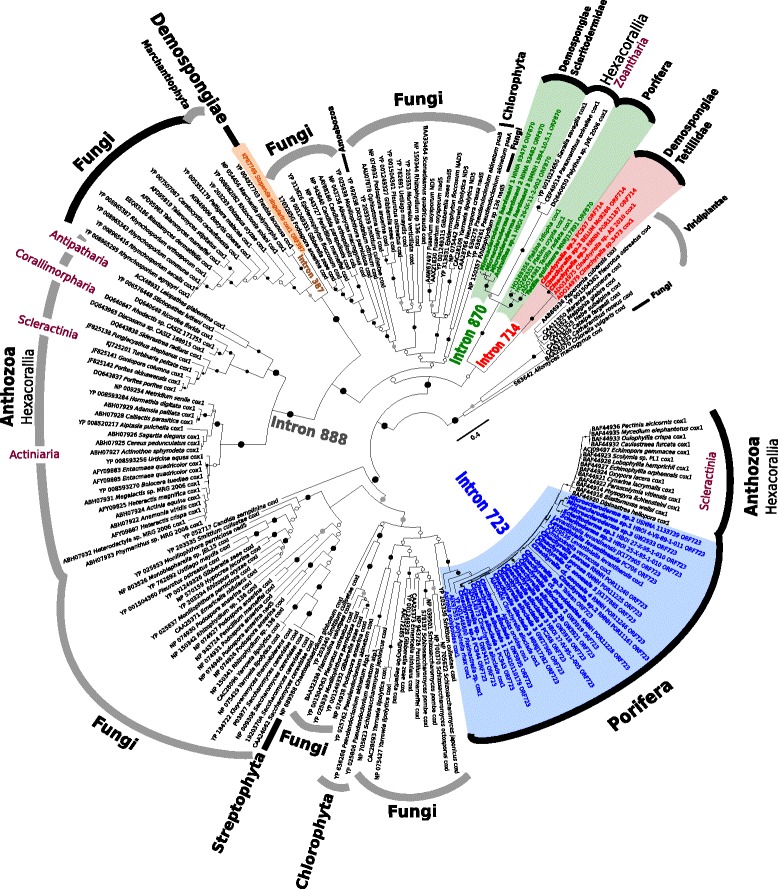
Maximum Likelihood phylogeny based on LAGLIDADG protein sequences of representative taxa from diverse groups. Circles on the branches indicate support values. *Black* circles: BP = 75–100. *Dark gray* circles: BP = 60–74. *White* circles: BP < 60. Numbers behind each taxa are GenBank accession numbers or voucher numbers. Sequences generated in this study are in bold. Sponge LAGLIDADG clades for introns 387, 714, 723 and 870 are indicated with *brown*, *red*, *blue* and *green* background respectively

The sponge LAGLIDADG sequences displayed phylogenetic affinities to four different clades (Fig. 7). LAGLIDADG (intron 387) of *Stupenda singularis* forms a highly supported sister group relationship with a Fungi/Marchantiophyta clade. Cnidarian LAGLIDADG encoding sequences are only present in Hexacorallia and never revealed in the other subclasses Octocorallia and Ceriantharia. Within Hexacorallia, the sequences of intron 888 are monophyletic and include the orders Actiniaria (sea anemones), Scleractinia (stony corals), Corallimorpharia (corallimorphs) and Antipatharia (black corals) [31]. Notably, there are no sponge LAGLIDADG (intron 888) sequences. In comparison, several scleractinian sequences form a clade with the sponge LAGLIDADG intron 723 sequences.

LAGLIDADG (intron 870) of *Plakina*/*Tetilla* and the Scleritodermidae are closely related to Hexacorallia (Zoantharia) LAGLIDADG sequences, but with considerable genetic distance. The genealogical affinities of intron 714 appear unresolved.

## Discussion

### Characterisation and mobility mechanisms of group I intron

**Fig. 8:**
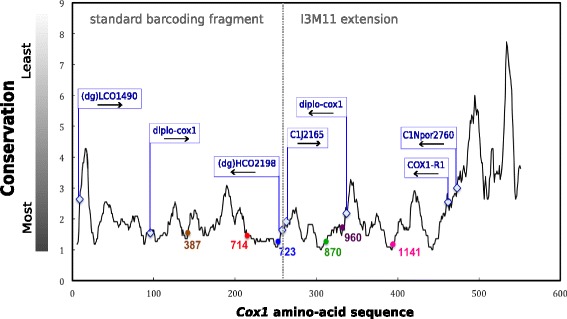
Conservation profile of the complete translated *cox1* gene for sponges. X-axis indicates the amino acid position along the alignment. Y-axis assigns the number of amino-acid substitutions over a range of 11 aligned positions throughout the alignment. Different intron insertion positions of group I introns (387, 714, 723 and 870) and group II introns (966 and 1141) are plotted on the profile line and color-coded with respect to Fig. 2. Additionally, the 5′ position of commonly used sponge barcoding markers is indicated as *light blue* rhombuses. A detailed list of primers is provided in Additional file 4

In sponges, group I and II introns occur coincidentally and exclusively in the *cox1* gene in a few sponge groups (Fig. 1). The current study specifically targeting sponge mitochondrial *cox1* introns represents the largest sponge mt intron dataset (95 specimens), encompassing 11 different sponge genera. In accordance with previous studies [21, 43, 47, 65, 82], double or single intron insertion sites within the *cox1* gene were observed among different taxa (Fig. 2). Sponge group I intron sequences in most cases contain a putative homing endonuclease (HE) ORF, which encode for a LAGLIDADG-type protein, unless they are degenerated (Fig. 2). Intron mobility is facilitated by those site specific HEs, which conduct double-strand breaks (DSBs) in alleles that lack introns, hence activating intron mobility via a DSB-repair process [2]. Those HEs are known to promote their mobility towards conserved regions [35]. However, only a few studies have investigated the conservation of those introns in their host genes. Swithers et al. [80] analysed the conservation of group I and group II introns in the host genes of vascular plants, protists, fungi, green algae, liverworts and amoeba, but not in animals. In the former, group I introns are preferentially located within conserved regions, whereby group II introns were not shown to remain particularly in conserved sites [80]. In comparison, our conservation analysis (Fig. 8) corroborated these findings by demonstrating that even in early branching metazoans like sponges, group I introns are located in the most conserved regions of their host proteins. At present, sponge group II intron insertions are only known from a single demosponge (*Cymbaxinella verrucosa*) [43], with two group II introns in the 3′ region of *cox1*. Our conservation analysis showed that these two group II introns were located in conserved regions (Fig. 8), however, additional data are needed for a better understanding of group II intron conservation and mobility in sponges.

The *cox1* gene in demosponges has the lowest substitution rate of all mt protein coding genes [90]. In fact, the mitochondrial genomes of the sponge classes Homoscleromorpha and Demospongiae possess features shared with non-metazoan opisthokonts rather than Bilateria such as the presence of intergenic regions, genes of foreign origin, a low substitution rate, selfish elements and introns [50]. Mt introns are also found in plants [28], fungi [44], Placozoa [76], and Hexacorallia [13, 37, 74] all known to have slow rates of evolution. It is assumed that this lower substitution rate slows down the elimination of ribozyme activities within group I introns, therefore HEGs would degenerate slower in most fungi [23], anthozoans [27, 31] and placozoans [9]. Intron mobility is particularly dependant on secondary structure and therefore mutation pressure, so sponge introns survive in the most conserved mt gene (*cox1*), and the most conserved regions of this gene (Fig. 8), where their HEG is most likely to degenerate slowly. On the other hand, hexactinellid and particularly calcareous sponges possess an accelerated substitution rate [51, 52] and no known mt introns to this date. This correlation between higher mutation rate and absence of mt intron is shared by Cubozoa [77], Ceriantharia [79]; Ctenophora [48, 59], Hydrozoa and Scyphozoa [42]. One exception to this pattern is the apparent lack of introns in Octocorallia, despite their lower substitution rates compared to the intron-bearing Hexacorallia [37, 74]. This might be due to the presence of a unique *MutS* gene, which encodes a DNA mismatch repair machinery [4], which prevents intron insertions. The mt DNA mismatch repair machinery in sponges remains unknown.

Sponge group I introns consist of complex catalytic ribozymes (RNAs) that fold into a conserved three-dimensional core structure of ten helices. Within this structure, the sponge HEGs are found to be always located within the loop region of the catalytic domain (helix P8, Fig. 6) as in Hexacorallia [31]. HEGs and their intron partners are thought to move either independently from each other [73] or as a single unit [34]. Whether those HEGs are actively expressed or not often depends on their functionality. The functional expression of HEG group I introns and the resulting gains and/or losses are considered as a cyclical process of different stages [30]. Emblem et al. [20] applied this into an evolutionary model for a group I intron in sea anemones and reported five stages: 1) Intron with HEG expressed and fused in frame with the upstream host gene exon; 2) Intron with expressed free-standing HEG; 3) Intron with shortened/degenerated HEG; 4) Intron without a conserved HEG and 5) Exon *cox1* without intron. Until now, only a few insights into this evolutionary model were given for sponges. Different stages are observed for intron 723 and intron 870 in different sponge species [43]. However, no detailed information has been provided yet on the potential start and stop codons, which are crucial diagnostic features for their categorisation. The potential start and stop codons, observed in all group I introns (Fig. 2), in addition to the predicted secondary structures (Fig. 6, Additional file 2), provide insights into the respective evolutionary stages of all sponge group I introns. In detail, we classified intron 387 of *Stupenda singularis* in stage 1. Intron 714 of all *Cinachyrella* sp. 2 appear in stage 1, and in stage 4 for *Plakinastrella* sp. due to several start and stop codons and no HEG. Intron 723 is found to be in stage 1 among all *Cinachyrella* species except *C.* sp. 2 (see below), which is in concordance with the already published data [43]. Intron 723 in *Microscleroderma* sp. 1 & 2 and *Scleritoderma* sp. 1 & 2 are also found to be in stage 1. A study comparing the length of DNA and RNA in combination with RT-PCR on a *Cinachyrella* intron 723 from Taiwan (probably *Cinachyrella* sp. 5) suggests that it can self-splice in vivo or in vitro [12]. It confirms that this particular stage 1 intron 723 is active. We observe intron 723 also in stages 3 and 4 in *Aplysinella rhax, Microscleroderma* sp. 3*, Cinachyrella* sp. 2, *Setidium* sp.1 (degenerated HEG) and *Aciculites* sp. 1 (short sequence and no HEG) respectively, which rebuts the suggested recent infection of intron 723 in sponges [43]. Intron 870 was at stage 1 for *Tetilla radiata*, *Plakina crypta* and *Plakina trilopha* [43] and now shown for *Microscleroderma herdmani* 1–3, *Tetilla quirimure* and *Setidium* sp. 1. Interestingly, both stage 3–4 intron 870 (*A. polypoides, A. oroides*) previously described [43] co-occur with stage 1 intron 723. Also, the only stage 4 intron 714 (*Plakinastrella* sp.) co-occurs with a stage 1 intron 723. Similarly, all of the stage 3–4 intron 723 (*Microscleroderma* sp. 3*, Cinachyrella* sp. 2*, Aciculites* sp. 1, *Setidium* sp. 1) co-occur with stage 1 introns (either 714 or 870). Overall, two stage 1 introns never co-occur, one of the two is always degenerating. We can therefore hypothesize that the presence of two group I introns is unstable or that maybe the degeneration of one somehow enables the insertion of a different intron. More double-intron-bearing *cox1* sequences are needed to study this further. Moreover, we noted that although Scleractinia (Hexacorallia) possesses intron 723 or 888, no evidence of double-intron *cox1* sequences in this group is given, which applies for Cnidaria in general. Since double-intron *cox1* sequences are also absent in Placozoa, sponges (e.g., demosponges and homoscleromorphs) are to date the only metazoans with double-intron *cox1* sequences.

### HGT versus VGT of group I introns

The sporadic detections and patchy distributions of group I introns not only among sponges, but also among other Metazoa in e.g., scleractinian corals [27, 31], plants [67] and fungi [45] are the main arguments for HGT.

**Fig. 9:**
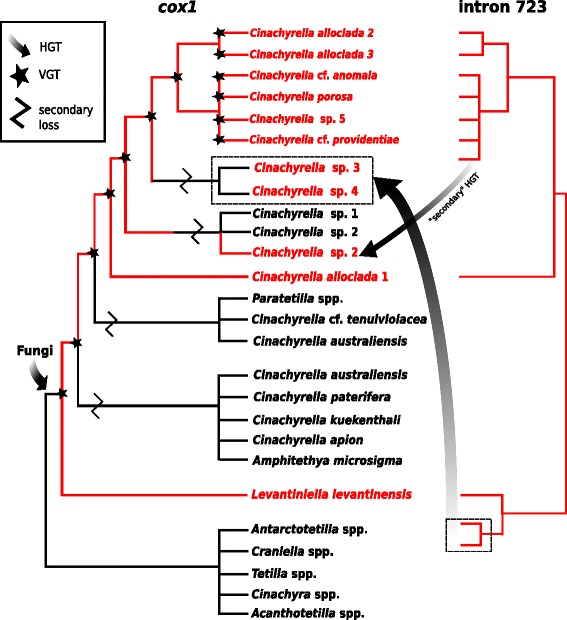
Comparison of the phylogenetic relationships of *cox1* and intron 723 illustrating HGT, VGT and secondary losses within the family Tetillidae

HGT events for group I introns in sponges were first hypothesized by Rot et al. [65] and later corroborated by other studies, based on major differences between *cox1* and intron phylogeny topologies [82] as well as the occurrence of homologous introns in phylogenetically distantly related sponge groups e.g., homoscleromorphs [90] and demosponges (Verongimorpha and Heteroscleromorpha) [21, 43, 47] (Fig. 1). Our results reveal new cases of intron HGT, this time within the *Cinachyrella* species (Fig. 9). As an example *Cinachyrella* sp. 3 (GW3412, Red Sea) and *Cinachyrella* sp. 4 (PC944, Morocco) are sister to *Cinachyrella alloclada* 2*–*3*/ Cinachyrella* spp. from the Pacific whereas their intron 723 are sister to the intron of *Levantiniella levantinensis* (Fig. 9)*.* One can therefore hypothesize that an intron 723 of *L. levantinensis* (or one of its ancestors) invaded the ancestor of *Cinachyrella* sp. 3 and *Cinachyrella* sp. 4. In addition, two *Cinachyrella* sp. 2 specimens from the Pacific (RMNH POR11161 and RMNH POR11187) group together with other conspecifics in the *cox1* phylogeny; however, their introns are closely related to *Cinachyrella* species from the Pacific. Therefore, we hypothesize that those two *Cinachyrella* sp. 2 specimens from marine lakes and mangroves were reinfested by intron 723 after it was primarily lost, which would indicate a “secondary” HGT between *C*. sp. 2 and the Pacific *Cinachyrella* spp. (Fig. 9). It also seems that these two specimens are losing their intron 723 again, which is at stage 3. These hypotheses are significantly corroborated by the SH-test (*p* < 0.01). Another HGT event was found within the family Sceritodermidae: while *Setidium* sp. 1 is sister to *Microscleroderma* taxa in the intron phylogeny (Fig. 5), it branches off first of all other Scleritodermidae taxa in the *cox1* phylogeny (Fig. 4). However, the *cox1* topology is poorly supported around *Setidium* sp., therefore this HGT event is less obvious than in the two previous cases. Although the introns 723 found in the Agelasida (*A. oroides* and *C. verrucosa*) are fairly divergent, the intron and the LAGLIDADG phylogenies both suggest that they are phylogenetically related to the *Cinachyrella/Levantiniella* introns. Indeed, the Agelasida introns share a more recent common ancestor with the *Cinachyrella/Levantiniella* introns, than with the Scleritodermidae (Fig. 5). A HGT from an ancestor of *Cinachyrella/Levantiniella* species to some Agelasida could account for this result. In all these examples it can therefore be hypothesised that a HGT occurred between distantly related sponge groups. Although the mechanism of intron HGT is unknown at this point, we noted that these donor/receiver species originate from the same regions and share the same habitats (reef, lake or mangrove, see Additional file 3), which is expected to make HGT possible.

Similarities in intron secondary structures of distantly related sponges are further evidence for HGT [43]. Hence, independent insertion events in Tetillidae, Axinellidae and Agelasida were proposed for intron 723 [43]. This is confirmed by secondary structure differences we observed in closely related families (Tetillidae and Scleritodermidae) (Fig. 6, Additional file 2). Additional loops (P9.1e,f), reduced stems (e.g., P9.1d) and the absence of the P6d region in Sceritodermidae (Fig. 6) result in a higher structure similarity to e.g., *Axinella polypoides* [43], rather than to other Tetillidae structures ([65]; Additional file 2, [82]), which confirms independent insertions of intron 723 in Scleritodermidae and Tetillidae. No major structural differences of intron 723 in the P9 and P6 regions were observed within different species of *Cinachyrella* (Additional file 2) except for *Cinachyrella* sp. 4 from Morocco, which showed reduced P9.1c and P9.1d helices. A few minor differences were also noted between *Cinachyrella* and *L. levantinensis* structures (Fig. 2 in [65]): the latter had an additional loop in P5a, a reduced P9.1d and a loop at the end of P6d. Interestingly, the latter two features are also observed in *C*. sp. 4 (Additional file 2), which could be explained by their common origin resulting from a HGT (Fig. 9). The relative similarity of intron 723 between *Cinachyrella* and *Levantiniella* is a strong argument in favor of a single insertion event in this clade, which therefore implies at least two losses of intron 723 to account for the two major *Cinachyrella/Paratetilla/Amphitethya* clades without any intron (Fig. 9). These would be the first reported cases of mt intron secondary loss in sponges.

For intron 870 no structure differences were observed between *Tetilla quirimure* (MNRJ 17891) (Fig. 6) and *Tetilla radiata* (HM032742) [82]. Remarkably, *Tetilla japonica* (JX177901), which is sister to *Tetilla radiata* (with a strongly supported node) does not posses intron 870. We therefore assume that *Tetilla japonica* secondarily lost intron 870, which would represent another case of mt intron loss in sponges. The structure of intron 870 in *Microscleroderma herdmani* 3 displays a reduced P6a and an additional P5d region (Fig. 6d) compared to *Tetilla radiata / quirimure,* which suggests an independent insertion of intron 870 as the most plausible explanation. This is further corroborated by the distant phylogenetic relationship between *Tetilla* and Scleritodermidae (Fig. 4) and the LAGLIDADG phylogeny (Fig. 7).

Until now VGT was only assumed within sponges [82], but awaited proof with a wider sampling. For the first time our study on 63 *Cinachyrella* sequences provides conclusive evidence that introns were vertically transmitted due to 1) mostly congruent *cox1* versus intron phylogenies and 2) similarity of secondary structures among closely related species. Introns 714, 723 and 870 have all undergone VGT, but this is especially apparent for intron 723 for which we have the largest sampling (Figs. 5 and 9). VGT for group I introns are also known e.g., from hexacorals (nad5-717 intron, [19]), but is often difficult to ascertain due to the patchy distribution of introns. To conclude, our results demonstrate that introns 714, 723 and 870 undergo VGT, HGT and secondary loss events, and that both VGT and HGT can occur within one genus (e.g., *Cinachyrella*) (Fig. 9).

### Origin of group I introns

The origins of group I introns has been debated for many eukaryotic organisms (e.g., [63]) including sponges [43]. Fungi are proposed as the primary donor of mt group I introns not only in plants (e.g., [14]), but also in cnidarians [31] and sponges [47, 65, 82]. Placozoa have also been suggested as possible donors in sponges, but only for intron 387 in one species [48]. Sponge-fungal associations, pivotal for such HGT, are well-known for sponges (e.g., [40, 71]). However, only little is known about the specific fungal lineages associated with intron-bearing sponge taxa. For *Cinachyrella*, however, deep sequencing analysis recently identified Ascomycota as a dominant fungal phylum (*Cinachyrella* cf. *australiensis* and *Cinachyrella* sp. from the China Sea [38]). This is corroborated by data for *Cinachyrella alloclada* from the Caribbean that showed that the cosmopolitan *Phoma* sp. (Ascomycota) is the dominant sponge-associated fungus, while nine more ascomycete species were found [5]. Indeed, in the reconstructed LAGLIDADG protein phylogeny (Fig. 7) all intron 723 sequences of *Cinachyrella* and other sponge taxa display a close relationship to ascomycete intron sequences, but also Viridiplantae (Chlorophyta and Streptophyta). The latter is not surprising, as intron of chlorophytes and Viridiplantae similarly have their origin from Ascomycota [53, 87]. The huge assemblage of group I introns described in fungi increases the chance of a HGT event. When metazoans and plants host one or two group I introns in their *cox1*, fungus like *Ophiocordyceps sinensis* contains 21 group I introns within its *cox1* region alone [54].

The non-bilaterian LAGLIDADG-protein sequence dataset (Fig. 7) identifies Scleractinia (stony corals) and Zoantharia (zoanthids) sequences as the only sequences respectively homologous to the sponge introns 723 and 870. Although this is not very well supported, our results show a sister relationship between LAGLIDADG in Scleritodermidae and Zoantharia, which suggests they may have contaminated each other (the direction of the HGT is unclear at this point); alternatively they were contaminated by the same donor, un-sequenced as of today. Because the Scleractinia intron 723 LAGLIDADG sequences are nested within the sponge sequences (Fig. 7), Fukami et al. [27] suggested two alternative scenarios: 1) Scleractinia and sponges have a similar fungi donor which independently transferred intron 723 in each group or 2) HGT events from each other (sponges to coral, or vice versa). However, although intron 723 has a patchy distribution in the Scleractinia [27], the Scleractinia LAGLIDADG sequences form a well supported clade (Fig. 7), which suggests that the origin and therefore the donor must have been the same. Different sponges can be excluded as donors, because otherwise the Scleractinia sequences would be partly mixed with the sponge sequences. Also, the possibility of one single sponge donor is unlikely, because there is no sponge species living in close contact with all these Scleractinia species from the Indo-Pacific and the Atlantic. Thus, we are in favor of a donor, most probably a fungus, which transferred intron 723 to different Scleractinia, while similar donors transferred intron 723 to different sponges. These donors can probably also act as vectors, thereby enabling HGT between *Cinachyrella* species, as shown above. Intron 387 of *Stupenda singularis* is the only sponge LAGLIDADG sequence apparently unrelated to any coral LAGLIDADG in the data set, but is closely related to LAGLIDADG found in fungi and Marchantiophyta (liverworts). Liverworts are thought to have received their introns from fungi [58], and the close relationship of the *Stupenda singularis* LAGLIDADG 387 likewise suggests a fungal origin (see also [47]).

Unfortunately, the origin of sponge intron 714 remains unresolved, since no supported relationship to any of the included taxonomic groups is given. Therefore, we cannot exclude the possibility that group I introns in sponges may originate from an undiscovered and/or un-sequenced sponge-associated symbionts, e.g., fungi, Archaea, Bacteria or dinoflagellates, since all are known to posses group I introns. In particular sponge bacterial symbionts, which can contribute to over 50% of the sponge biomass [39, 69], may play an essential role as potential intron donors. However, according to our results (Fig. 7) it is unlikely that bacteria, archaea or dinoflagellates are donors, because of the absence of a homologous LAGLIDADG motif. Blastx of intron 714 specified fungi as best hits, therefore fungi remains a good donor candidate for this intron. According to the diversity of habitats of intron-bearing sponges, from shallow water (in reefs, marine lakes and mangrove) to the deep sea (Additional file 3), we can hypothesize that the putative intron donor may also be ubiquitous, and present in all these different environments.

### Implications for DNA barcoding

Intron insertion in highly conserved *cox1* regions decreases the possibility of intron elimination, because the removal must be specific in order to avoid any disruption of the protein function. The most widespread intron 723 is located at the most conserved site in the *cox1* gene (Fig. 8), suggesting this position as an “intron hotspot”. In addition to the high number of intron 723 in Tetillidae, we discovered several more intron 714 (*Cinachyrella*) and intron 870 (Scleritodermidae, *Tetilla*), both in conserved *cox1* regions. Intron presence in conserved *cox1* gene regions has major consequences for other fields of science such as molecular taxonomy. In order to gain a better understanding of locations and conservation of the currently recommended barcoding primers [22, 24, 91], we plotted the 5′ site of each primer on the *cox1* conservation profile line (Fig. 8). Interestingly, for the standard barcoding fragment and the I3M11 extension our analysis shows that all previously applied sponge barcoding primers are located in comparatively less conserved regions (Fig. 8). Moreover, our results indicate that group I intron 723 and group II intron 960 are in close proximity to barcoding reverse primer sites (HCO2198 and diplo-cox1) or interrupt the priming regions (intron 723), which corroborates earlier findings from Szitenberg (2010). These findings may partly explain the low (~25% mean) amplification success reported for barcoding museum samples using standard barcoding primers [86]. We therefore recommend for future sponge barcoding studies to test the reverse COX1-R1 primer, which is more distant to the intron insertions, instead of the HCO2198 primer (Fig. 8). The COX1-R1 primer, originally designed to amplify the *cox1* of Tetillidae [65], has been shown to successfully amplify *cox1* in Poecilosclerida [62], Agelasida and Axinellida [43], Chondrosiida and Dictyoceratida [3], some Astrophorina (P. Cárdenas, unpublished results) and Spirophorina (this study).

## Conclusion

This study provides novel insights into the taxonomic distribution, diversity and mobility of mitochondrial group I introns in sponges, and validates the subclass Spirophorina (Tetractinellida), as an *intron hotspot* in sponges, notably by increasing the number of Tetillidae introns known by a factor of 5. We wonder whether this could be linked to a lower mt mutation rate in the Spirophorina with respect to other sponges, as suggested for some intron hotspot fungi groups [44]. We show that co-occurrence of two introns in *cox1* is unique among metazoans, but not uncommon in sponges. However, this combination always associates a potentially active intron with a degenerating one. Earlier hypotheses of HGT were confirmed and for the first time VGT and secondary losses of introns conclusively demonstrated. Consequently, such a high level of HGT in combination with the relative low variation in case of VGT (e.g., intron 723, Fig. 9), rejects any alternative use of mt introns as phylogeographic markers. Since the majority of sponge introns encode a HEG in frame with the 5′ exon, activity of those introns is assumed. We further demonstrate that introns are not restricted to shallow water sponge species, but also occur in species from deeper (~500 m) habitats and extreme environments (mangroves and marine lakes). Conservation profile analysis reveals that all group I and possibly also group II intron insertions in sponges are located within the most conserved regions of their host protein, which may partly explain why they persist in their host genes. At the same time, we show that the currently used sponge barcoding primers are usually located in less conserved regions compared to the introns, but can also overlay intron insertion sites. Therefore, we recommend applying different primers (in particular reverse primers) when standard barcoding primers fail to amplify the *cox1* gene. Finally, our study enhances the support for a fungal origin for the majority of introns in sponges.

## Methods

### Sampling and identification of specimens

*Cinachyrella* samples were collected in Florida (U.S.A.) by snorkeling in the seagrass meadows adjacent to the Mote Marine Laboratory/Tropical Research Laboratory (Summerland Key, Florida U.S.A.) and by scuba-diving on the Broward County reef located off Fort Lauderdale (26° 10.498, −80° 05.632). More *Cinachyrella* spp. were collected in Indonesia by diving on reefs and snorkeling in mangroves and marine lakes in West Papua and East Kalimantan, Indonesia. The remaining material was obtained through collaborators or sampled in several museum collections (Additional file 3). Because of ambiguous sequences or missing data, some *Cinachyrella* specimens from Szitenberg et al. [81] were successfully re-sequenced (JX177885, JX177886, JX177887 and JX177913). Taxonomic identification to genus and species level was performed by the authors and follows the findings of Carella et al. [11] on Tetillidae. The species *Craniella quirimure* from Brazil was re-assigned to the genus *Tetilla* based on the absence of a clear double-layered cortex. In some cases identification of species was adopted from collections and earlier publications. Numbers were added for lineages of species that could not be recovered as monophyletic and await revision (e.g., *C. alloclada* 1–3). A detailed list of species origin including collector, voucher numbers and accession numbers, location and depth are provided in Additional file 3. A *Cinachyrella cox1* sequence (including a group I intron) from Taiwan was manually copied from Hsiao [41]. This species was first identified as *Cinachyrella australiensis* and is identified as *Cinachyrella* sp. 5 based on our *cox1* CDS phylogeny. One complete *cox1* sequence of *Microscleroderma* sp. (USNM 1133739) with an intron was kindly provided by D. V. Lavrov (Department of Ecology, Evolution, and Organismal Biology, Iowa State University, USA). The higher level demosponge classification follows Morrow & Cárdenas [56].

### Molecular approach

Genomic DNA was isolated from the choanosome of the sponge tissue by using the NucleoSpin (Machery-Nagel) or the DNeasy (Qiagen) Blood and Tissue Kit according to the manufacturer’s protocol. An additional centrifugation step was added before transferring the lysate to the Spin Column in order to avoid any clogging of the membrane, caused by sponge spicules. Quantification of the isolated genomic DNA was performed using a NanoDrop 1000 Spectrophotometer (Thermo Scientific).

Amplification of the partial *cox1* was performed by using different primers and PCR conditions. Detailed information of primers used for each sample is provided in Additional file 4. For most Tetillidae the *cox1* fragment was amplified using the primers LCO1490 [24] and COX1-R1 [65] and for most Scleritodermidae we used the primers diplo-cox1-f1 and diplo-cox1-r1 [52]. For both primer pairs the PCR settings were: 94 °C, 5 min; (94 °C, 1 min; 50–52 °C, 1:30 min; 72 °C, 1:30 min) × 40 cycles; 72 °C, 10 min. Amplified fragments were visually checked for introns by length on a 1.5% agarose gel. For the majority of the *Cinachyrella* samples with introns, we observed an additional non-specific band at position ~600 bp of bacteria and fungi *cox1* fragments. Separation of double bands and PCR clean-up was performed using a modified freeze-squeeze method [83] in which 20 μl of the PCR product were cut from the gel and stored at −80 °C for one hour, followed by a 40 min centrifugation step at 14,000 rpm. The supernatant (6 μl) was used for cycle sequencing with different and multiple sequencing primers (Additional file 4) together with BigDye Terminator v3.1 (Applied Biosystems, Forster City, CA, USA) chemicals and sequenced by an ABI 3730 Genetic Analyzer at the Sequencing Service of the Department of Biology (LMU München), or by Macrogen (South Korea).

### Positions and secondary structures of group I introns within Tetractinellida

Insertion sites for each intron were ascertained in an alignment including other intron-bearing sequences [21, 43, 82]. Intron specific positions were defined according to the *cox1* sequence of the sponge *Amphimedon queenslandica* following Szitenberg et al. [82]. Blast hits and sequence similarity to already published group I intron insertions were used to distinguish between different insertion sites and group I and group II introns. An overview of the different group I (intron 387, 714, 723, 870) insertion sites as well as group II (intron 966 & 1141) insertion sites is given in Fig. 2. Identification of the HEG for each ORF was conducted by blastp against NCBI Genbank [1]. The class of group I introns (IA, IB, IC, ID or IE) was obtained using the RNAweasel Website http://megasun.bch.umontreal.ca/RNAweasel/ [49]. Initiation and stop codons of the HEG ORFs were located using the ORF finder as implemented in Geneious v.8.1.8 (www.geneious.com) with the following settings: translation Table 4 (Mold and Protozoan mitochondrial) with start codons ATG, GTG, TTG and ATT [90], minimum size 100 bp, including interior ORFs. Although considered as potential start codon in sponge group I introns [47, 65], there is actually no evidence that TTA is used in sponges as start codon; it has only been found so far in *Trypanosoma* [26]. We therefore excluded the TTA start codon in our searches and also revisited the ORFs of previously reported sponge group I introns.

In order to predict the secondary structures of group I introns, we manually converted the given secondary core structures into a dot-bracket notation including pseudoknot informations in square brackets. As secondary structure references, we used *Cinachyrella alloclada* (HM032738) for intron 723 and *Tetilla radiata* (HM032742) for intron 870 [82]. In order to ensure the right structure annotation for short variable (mainly P6 and P9) domains, we used Mfold http://unafold.rna.albany.edu/ [93] under the general settings, presupposing the exclusion of additional pseudoknots, which cannot be predicted by this program. Those Mfold structures were then manually converted into a dot-bracket notation and implemented to the already established core structure sequence. SeaView v4 [32] was used to align the sequences to their structure annotation. The LAGLIDADG regions were removed from the sequences for further analysis. The rest of the intron sequence together with its structure information, was converted to a ct-format using the Perl-script (2ct.zip) of Voigt et al. [89] (available at http://www.palaeontologie.geo.lmu.de/molpal/RRNA/index.htm). All secondary structures were visualized in RNAViz 2.0.3 http://rnaviz.sourceforge.net/ [18]. Helix names follow Szitenberg et al. [82].

### Tetractinellida phylogenies predicted by *cox1* CDS

#### Sequence alignments and outgroup choice

Newly generated sequences as well as additional GenBank sequences were manually aligned to the datasets from Szitenberg et al. [81, 82]. Aligned sequences were subsequently controlled for discrepancies and corrected by eye. Two Astrophorina species (*Geodia barretti* and *Alectona millari*) were used as outgroups in Tetillidae phylogenetic analyses. Astrophorina has been established as the sister clade of Spirophorina in previous studies [6, 57]. For the analysis of the tetractinellid phylogeny we chose *Halichondria melanodocia* and *Axinella corrugata,* which were already successfully used as outgroups in previous studies on the molecular phylogeny of the Tetractinellida (e.g., [47]).

The final *cox1* alignment (excluding intron(s)) of the Tetillidae phylogeny comprised 133 sequences (including the two outgroups), of which 76 were newly generated from this study. The alignment was 1177 bp long, of which 829 bp were constant, 62 bp were parsimony uninformative and 286 bp were parsimony informative. The final *cox1* alignment of the Tetractinellida phylogeny constituted 82 sequences (including the two outgroups) of which 33 were newly generated from this study. In total the alignment comprised 1118 bp, of which 642 bp were invariant, 77 bp parsimony uninformative and 399 bp were parsimony informative.

#### Phylogenetic reconstructions

Phylogenetic tree reconstructions for both analyses were performed on a parallel version of MrBayes v3.2.4 [64] and RAxML v8.0.26 [78] on a Linux cluster. Bayesian analyses were conducted under the most generalized GTR + G + I evolutionary model, as resulted from jModelTest v.2.1.7 [16]. Analyses were run in two concurrent runs of four Metropolis-coupled Markov-chains (MCMC) for 100,000,000 generations and stopped when the average standard deviation of split frequencies reached below 0.01. The first 25% (burn-in) of the sampled trees were removed for further analysis. For both datasets, Maximum Likelihood (ML) and bootstrap analyses (1,000 replicates) under the GTR + G model as resulted from jModelTest v.2.1.7 [16] were performed. Tree topologies from Bayesian and ML analyses were compared and visualized using Figtree v1.4.2 http://tree.bio.ed.ac.uk/software/figtree/.

### Phylogenetic inference based on intron + LAGLIDADG sequences

In order to test for vertical transmission of group I introns (including both LAGLIDADG and the non-coding regions) in the genus *Cinachyrella*, we conducted phylogenetic analyses on separate datasets respectively including all sponge introns 723, 714 and 870. For the analysis of intron 723, we included 74 sequences of which 63 belong to the genus *Cinachyrella*. One taxon *Aciculites* sp. 1 (HBOI 26-IX-11-2-002), was excluded from this analysis, as no putative HEG were detected in the intron. The final intron 723 alignment was 1167 bp long, of which 488 bp were constant, 307 bp parsimony uninformative and 372 bp parsimony informative. The final intron 714 dataset included 13 sequences and was 946 bp long, of which 891 bp were constant, 49 bp were parsimony uninformative and 6 bp were parsimony informative. As an outgroup for both analysis we used the introns of *Plakinastrella* sp. (NC 010217), a species that belongs to a different sponge class (Fig. 1). The final alignment of intron 870 contained 12 taxa and was 974 bp long, of which 615 pb were constant, 46 bp were parsimony uninformative and 313 bp parsimony informative. *Plakina trilopha* (HQ269356) and *P. crypta* (HQ269352) which belong to a different sponge class (Fig. 1) were used as outgroups. Phylogenetic tree reconstructions were performed as described above for the *cox1* exon phylogeny.

In order to test whether the incongruencies between the exon and the intron/HE phylogeny were significant, we performed a series of Shimodaira-Hasegawa (SH) tests [75] as implemented in RAxML [78] on the exon tree against ML topologies constrained towards the intron tree topology. Constraints were inferred with Mesquite v.3.10 [55].

### Phylogenetic reconstructions based on LAGLIDADG protein sequences of group I introns

In order to investigate the evolutionary origins of the putative LAGLIDADG encoding introns (387, 714, 723 and 870) in sponges the newly generated sequences were added to the LAGLIDADG dataset by Huchon et al. [43]. Additionally, we included 12 fungal and two Marchantiophyta LAGLIDADG sequences resulting from Blastp hits of the sponge LAGLIDADG for intron 387 (Table 2, [47]). Subsequently, MAFFT v.7 [46] under the L-INS-I algorithm was used to generate the protein alignment. The resulting alignment contains sequences of fungi, plants, cnidarians and sponges. Here, 291 amino-acids (aa) out of 1278 aa were parsimony-uninformative variable characters, 729 aa were constant and 708 parsimony-informative. As a result, we manually corrected the LAGLIDADG alignment. Parts with more than approximately 50% of missing data were removed manually using the custom site set selection tool in SeaView. The final alignment was 317 amino-acids long, of which one character was constant and two variable characters were parsimony-uninformative. The rest of the 314 characters were phylogenetically informative. The maximum likelihood (ML) analysis was performed using RAxML v8.0.26 [78] on a Linux cluster with 1,000 bootstrap repeats. Using ProtTest 3.4 [15] the best evolutionary model was found to be VT + I + Gamma + F. However, for the RAxML analysis we excluded the invariant parameter (I) from the model, as it is not recommended to use both gamma (G) and invariant (I) parameters among site-rate variations according to the RAxML manual. No root for the tree was specified, as it was not needed for our purpose.

### Compilation of the conservation profile

A conservation profile was calculated from a *cox1* protein alignment dataset compiled from the demosponge sequences from [90], complemented by Homoscleromorpha sequences from [29]. The final protein alignment consisted of 58 sequences and 556 characters. The conservation profile was made following Swithers et al. [80] using the same perl script (made available in the supplementary material of Swithers et al. [80]) but with a slightly modification to allow ‘X’ characters in the alignment and calculation. The 5′ position of the common barcoding markers as well as all sponge intron insertion positions were plotted on the profile line.

## Additional files

Additional file 1: Intron 714 and 870 phylogenies. (PDF 38 kb)
Additional file 2: Predicted secondary structure of introns from different *Cinachyrella* species. (PDF 86 kb)
Additional file 3: Metadata of samples used in this study. (XLSX 85 kb)
Additional file 4: PCR and sequencing primers used in this study. (XLS 80 kb)
