# Supplementary figures and images for: Evolution of group I introns in Porifera: new evidence for intron mobility and implications for DNA barcoding
Source: BMC Evol Biol. 2017 Mar 20;17:82. doi: 10.1186/s12862-017-0928-9 (PMC5360047; doi:10.1186/s12862-017-0928-9)

### Intron+LAGLIDADG 870 Phylogeny

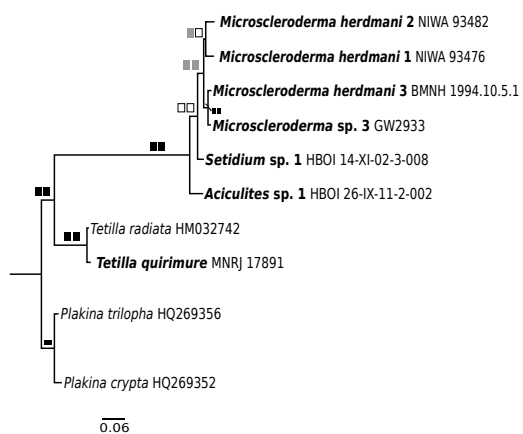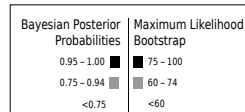

### Intron+LAGLIDAG 714 Phylogeny

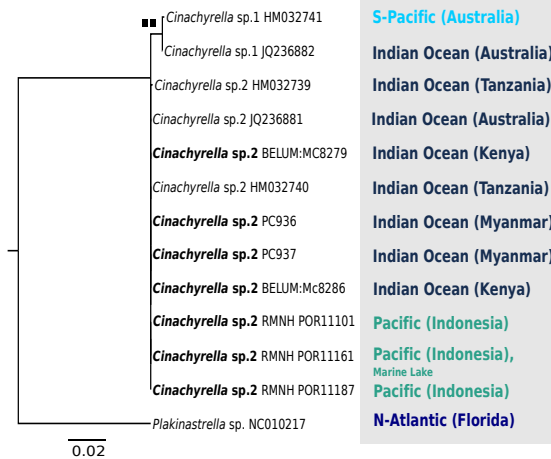

Supplement: Additional file 1: — Intron 714 and 870 phylogenies. (PDF 38 kb) [file 12862_2017_928_MOESM1_ESM.pdf]

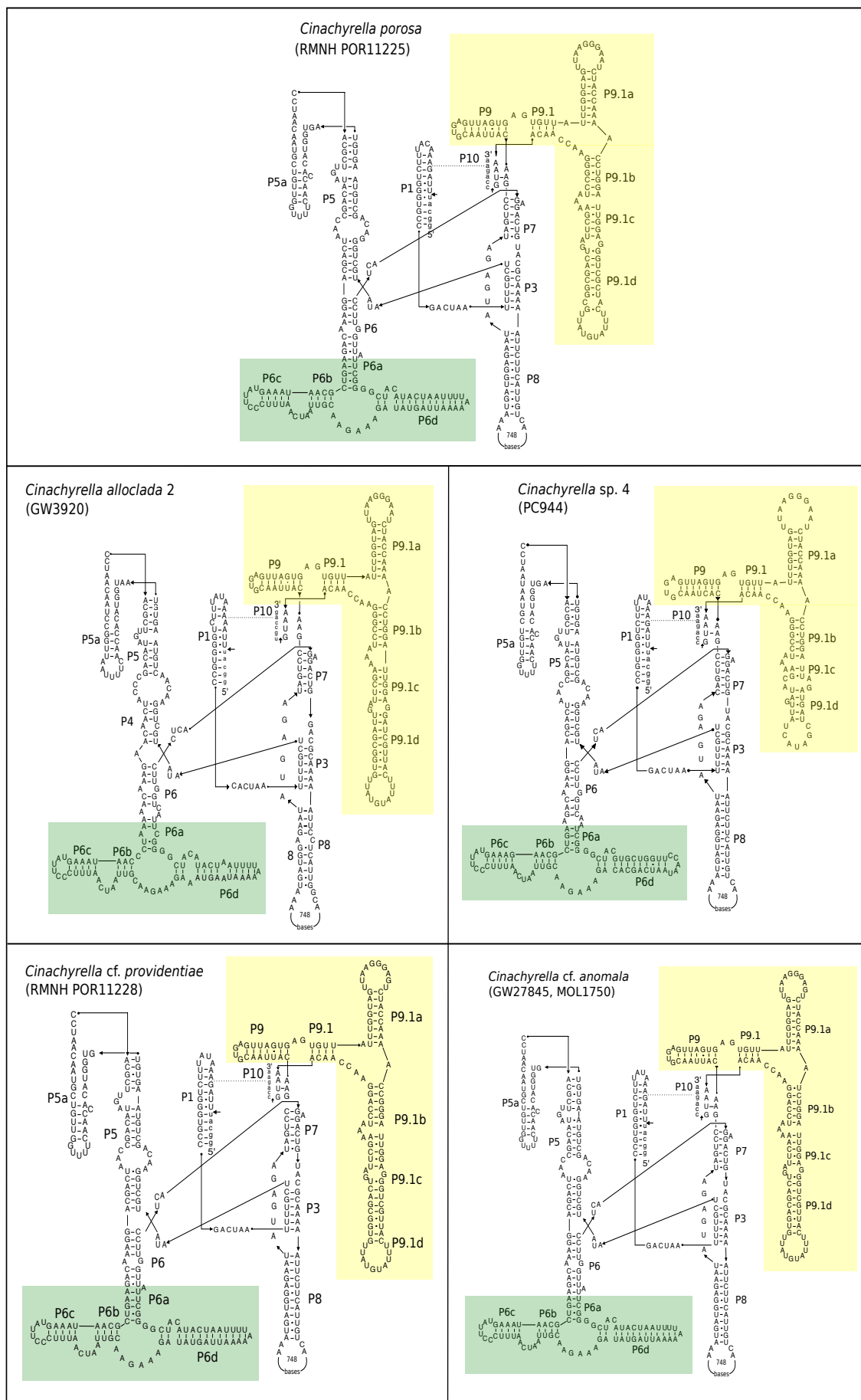

**Suppl. 2:** Predicted secondary structure of different *Cinachyrella* species

Supplement: Additional file 2: — Predicted secondary structure of introns from different Cinachyrella species. (PDF 86 kb) [file 12862_2017_928_MOESM2_ESM.pdf]
